# Reduction of inter-observer differences in the delineation of the target in spinal metastases SBRT using an automatic contouring dedicated system

**DOI:** 10.1186/s13014-021-01924-0

**Published:** 2021-10-09

**Authors:** Niccolò Giaj-Levra, Vanessa Figlia, Francesco Cuccia, Rosario Mazzola, Luca Nicosia, Francesco Ricchetti, Michele Rigo, Giorgio Attinà, Claudio Vitale, Gianluisa Sicignano, Antonio De Simone, Stefania Naccarato, Ruggero Ruggieri, Filippo Alongi

**Affiliations:** 1 Advanced Radiation Oncology Department, Sacro Cuore Don Calabria Hospital, IRCCS Ospedale Sacro Cuore Don Calabria, Via Don A.Sempreboni 5, 37124 Negrar Di Valpolicella, VR Italy; 2 University of Brescia, Brescia, Italy

**Keywords:** Radiotherapy, Stereotactic, Software, Spine, Metastases

## Abstract

**Background:**

Approximately one third of cancer patients will develop spinal metastases, that can be associated with back pain, neurological symptoms and deterioration in performance status. Stereotactic radiosurgery (SRS) and stereotactic body radiotherapy (SBRT) have been offered in clinical practice mainly for the management of oligometastatic and oligoprogressive patients, allowing the prescription of high total dose delivered in one or few sessions to small target volumes, minimizing the dose exposure of normal tissues. Due to the high delivered doses and the proximity of critical organs at risk (OAR) such as the spinal cord, the correct definition of the treatment volume becomes even more important in SBRT treatment, thus making it necessary to standardize the method of target definition and contouring, through the adoption of specific guidelines and specific automatic contouring tools. An automatic target contouring system for spine SBRT is useful to reduce inter-observer differences in target definition. In this study, an automatic contouring tool was evaluated.

**Methods:**

Simulation CT scans and MRI data of 20 patients with spinal metastases were evaluated. To evaluate the advantage of the automatic target contouring tool (Elements SmartBrush Spine), which uses the identification of different densities within the target vertebra, we evaluated the agreement of the contours of 20 spinal target (2 cervical, 9 dorsal and 9 lumbar column), outlined by three independent observers using the automatic tool compared to the contours obtained manually, and measured by DICE similarity coefficient.

**Results:**

The agreement of GTV contours outlined by independent operators was superior with the use of the automatic contour tool compared to manually outlined contours (mean DICE coefficient 0.75 vs 0.57, *p* = 0.048).

**Conclusions:**

The dedicated contouring tool allows greater precision and reduction of inter-observer differences in the delineation of the target in SBRT spines. Thus, the evaluated system could be useful in the setting of spinal SBRT to reduce uncertainties of contouring increasing the level of precision on target delivered doses.

## Introduction

Local back pain and neurological symptoms are the most common clinical disease presentations in spinal metastases [1]. Traditionally, surgical decompression and palliative radiotherapy have been recognized as the main treatment options for these patients. Nevertheless, both oncological approaches were associated with a limited local control probability [2] and pain relief [3]. Nonetheless, radiation therapy is still considered one of the important therapeutic options in the management of spinal metastases. In fact, in the last decades, significant non-invasive technological improvements have been observed in modern radiotherapy. Accurate radiological staging, as magnetic resonance imaging (MRI) and metabolic images (PET-CT), allows to detect any metastatic disease presentation earlier. Moreover, in the oligometastatic and oligoprogressive patients, it is progressively becoming current clinical practice to prescribe local ablative radiation treatments (SBRT) [4–6]. The combination of both strategies has the purpose to prescribe earlier ablative treatments, prevent local symptoms and postpone the prescription of new systemic treatments. In fact, SBRT allows prescribing in a limited number of fractions, high dose irradiation to small target volumes, and minimizing the dose exposure to organ at risks (OARs) [7–9]. This favorable characteristic is crucial in spinal metastases, in which an appropriate target volume definition is mandatory to avoid tumor missing, without compromising tumor coverage. For this reason, in the radiation oncology community, a standardized method in target definition is advocated, through specific guidelines and the development of contouring tools [10, 11]. As previously reported [12], a dedicated software has been developed for patients eligible to SBRT in spinal metastases (Elements Spine SRS®, Brainlab™ Germany). The preliminary results reported an excellent local control probability and a good toxicity profile. Despite this, the possibility to use an automatic target contouring tool (Elements SmartBrush Spine; hereby referred to as SmartBrush) could potentially support radiation oncologists to uniform the outline process. In fact, the principal advantage of the *SmartBrush tool* is represented by the identification of different density grey levels, which can decrease the inter-observer differences in target definition. The aim of the present study is to assess the SmartBrush impact in patients with spinal metastases, eligible for SBRT.

## Materials and methods

### Patients and treatment

In the current study, we analyzed the impact of the *SmartBrush* software (Elements SmartBrush Spine, Brainlab™ Germany) in patients with spinal metastases. Specifically, 20 spinal lesions eligible for SBRT treatments were included in the study. In all cases, 20 target volumes were equally visible and delineable both in CT scans (as osteolytic or thickening areas) and in MRI scans. All the procedures have been described in a previous publication [12]. Briefly, patient simulation was performed by acquiring a 1 mm-slice thickness CT scan with the aid of a head or abdomen thermoplastic mask, depending on the treatment site. The field of view was adjusted to cover the whole body along the entire tract of interest of the column.

For MRI scans, a T2-weighted sequence was rigidly registered with the simulation CT scan for a refined definition of healthy structures such as spinal cord, sacral plexus or cauda equina. The organs at risk (OARs), depending on tumor site, were automatically delineated by the software based on an anatomical atlas.

For baseline treatment, the gross tumor volume (GTV) was defined as the macroscopic contrast-enhancing lesion detected on diagnostic imaging; Smartbrush software allows an easier recognition of the affected region with a direct adjustment for CTV refined definition. No GTV manual refinement is performed. The clinical target volume (CTV) was automatically generated by the software according to international guidelines and automatically labeled to avoid vertebral crossing [11]. Afterwards, the CTV was cropped where adjacent to the spinal canal. The planning target volume (PTV) was obtained by adding an isotropic margin of 2 mm to the CTV, avoiding any potential overlap with the spinal canal. Prescribed dose and fractionation were chosen based on the tumor volume, previous spinal radiation treatment and OAR tolerance limits. Corticosteroid therapy was prescribed only if patient reported pain or any neurological symptoms.

To evaluate the impact of the automatic target contouring tool (SmartBrush), which uses the identification of different densities within the target vertebra, we evaluated the agreement of the contours of GTV of 20 spinal targets (2 cervical, 9 dorsal and 9 lumbar column) outlined by three independent observers experienced in spine SBRT treatments, using the automatic tool compared to the contours obtained manually and measured by DICE similarity coefficient.

Given the different contouring by independent observers as A and B, the Dice coefficient is defined as:

$$C_{dic} = \frac{{2\left| {A \cap B} \right|}}{\left| A \right| + \left| B \right|}.$$

The Dice coefficient represents the ratio of overlapped region between different contoured target volumes (0 ≤ C_dic_ ≤ 1).

The maximum value of C_dic_ is 1 when the volumes are identical, and the minimum value is 0 when the contours are fully totally dissimilar. This index has the properties of a metric system and it has been extensively used to quantify contouring agreement among observers. We did not consider it relevant to evaluate the variations of the CTV as they are dependent, according to the guidelines of International Spine Radiosurgery Consortium [11], more on the localization of the GTV within the vertebra, than on its extension and therefore scarcely modifiable by small variations of the GTV volume.

### Statistical analysis

To evaluate the performance difference, a Student’s t-test for two independent samples was used by assuming a normal distribution for the obtained metric values. A significance level of 5% was considered to show a statistically significant difference between the performances of two contouring tool (SmartBrush versus manual contouring).

## Results

Data from 20 patients treated for spine metastases were extracted for the purpose of the present analysis. More specifically, 2 were cervical targets, 9 thoracic and 9 were located in the lumbar spine. A median prescription dose of 24 Gy was delivered in 3 fractions using image-guided volumetric modulated arc therapy by means of a TrueBeam linear accelerator (Varian Medical System PA, California). A simultaneous integrated boost to the GTV was delivered for a median prescription dose of 27 Gy in 3 fractions.

**Fig. 1:**
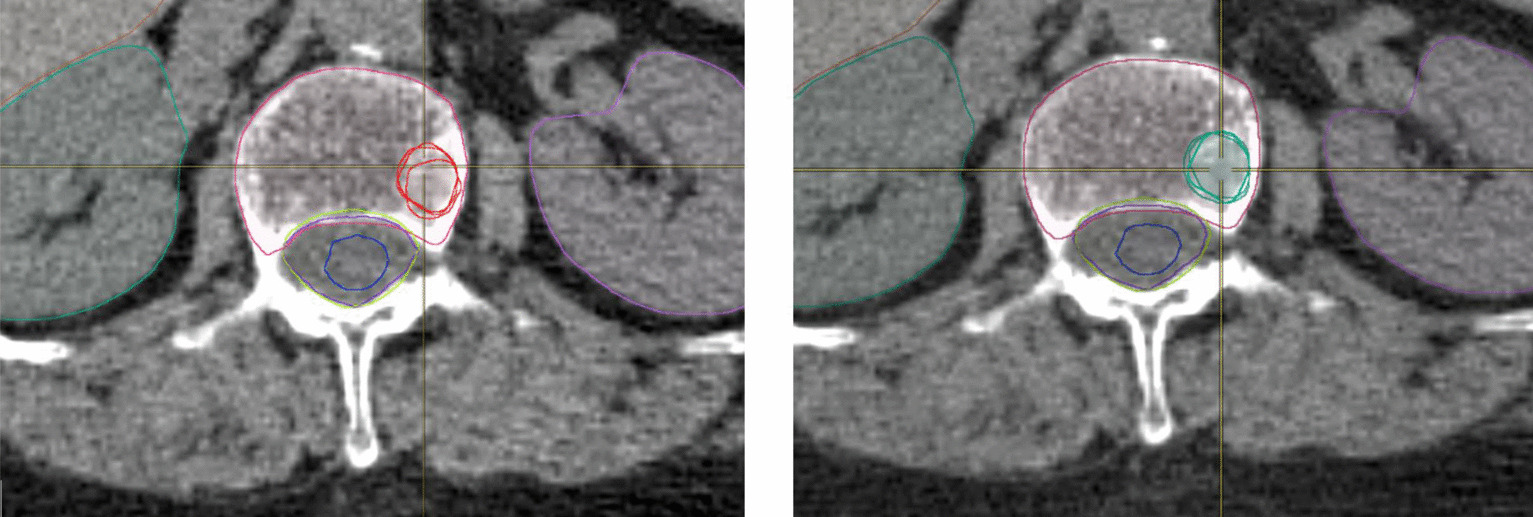
Axial images examples of GTV delineation by manually method and Smart Brush Tool. Red: Manual contouring. Green: Smart Brush contouring

Median GTV volume was 1.44 cc (range, 0.35–18.8), median CTV volume was 22.1 cc (range, 13.6–28.9). Examples of target volume delineation are displayed in Fig. 1.

**Fig. 2:**
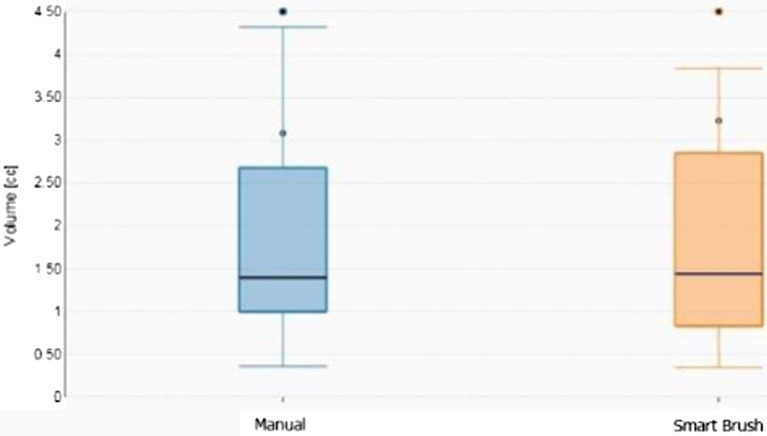
Boxplot of contour volumes per modality. Turquoise: Manual contouring. Orange: SmartBrush contouring

The agreement of GTV contours outlined by three independent operators was statistically superior (*p* = 0.048) with the use of the automatic contour tool (SmartBrush), that registered a mean DICE coefficient of 0.75 (with 95% confidence interval of 0.71–0.79) compared to manually outlined contours, that registered a mean DICE coefficient of 0.57 (95% confidence interval of 0.53–0.62), Fig. 2.

## Discussion

Radiation therapy represents a consolidate highly effective local approach for patients with spinal metastases, due to its potential cytoreductive effects on local disease to prevent possible neurological deterioration and its ability to relieve pain. In recent decades, spinal SBRT is emerging as an innovative and effective technique in the local control of spinal metastases in oligometastatic patients, for its capacity of delivering high radiation doses, potentially improving local control rates, as reported in patients with brain and extracranial metastases and early-stage non-small cell lung cancer [13, 14].

In recent years, it has been shown that the use of SBRT in spinal metastases was associated with a faster and improved pain response compared to conventional fractionated palliative radiotherapy, as demonstrated by a recent randomized phase II trial [15]. In different prospective trials and literature review, SBRT proves to be an effective and safe treatment option for spinal metastases, with a limited risk of complications, including vertebral compression fracture, flair pain and symptomatic myelopathy [16, 17], as also shown in our previous experience [12]. The results of a randomized phase II/III study have just been presented at ASTRO's Annual Meeting, comparing 24 Gy in 2 stereotactic body radiotherapy (SBRT) fractions versus 20 Gy in 5 conventional palliative radiotherapy (CRT) fractions for patients with painful spinal metastases, confirming the advantage of stereotactic treatment of spinal metastases [18].

For a spinal SBRT approach, even before the accurate administration of the high-dose treatment, allowed by the use of Image-Guided Radiotherapy systems, the correct definition of the target is mandatory for the identification of vertebral lesions and delineation of the organs risk present in the treatment field, allowed by the use of multimodal imaging.

In addition of this, the correct definition of the treatment volume becomes even more important in SBRT treatments, due to the high doses that are delivered and the proximity of critical OARs such as the spinal cord, thus making it necessary to standardize the method of target definition and contouring, through the adoption of specific guidelines and specific automatic contouring tools [10, 11].

The present study examines a specific contouring tool (SmartBrush) of a dedicated software (Elements®, Brainlab™ Germany) for spinal metastases SBRT. We evaluated the accuracy of this novel specific contouring tool -SmartBrush- applied to retrospective simulation CT clinically acquired in 20 patients treated with Spine SBRT.

To evaluate the advantage of using an automatic contouring tool (SmartBrush) based on the recognition of areas of different density within the target vertebra, we evaluated the concordance of the contours obtained by three independent observers with and without the use of the SmartBrush tool.

The results showed a statistically greater agreement in the use of the contouring tool analyzed, compared to the manual delineation of the contours, allowing for greater homogeneity in the definition of the target.

This last aspect is more relevant in the accurate definition of the GTV volumes, especially in view of the possibility of performing treatments with simultaneous integrated boost on the lesion evident in PET or MRI, within the vertebral target, to potentially increase local disease control.

To date, few experiences in the literature report on the use of softwares for automatic segmentation of the target in radiotherapy. Hearn et al. have recently published the results of a comparison study between manual segmentation and semi-automated delineation of prostate GTV based on quantitative thresholding of intraprostatic apparent diffusion coefficient (ADC) and PET standardized uptake values (SUV), recording a significantly higher inter-observer agreement with the use of PET compared to MRI-based semiautomatic segmentation [19].

Autosegmentation tools are an attractive option for clinicians, since they are supposed to facilitate everyday clinical practice, by reducing inter-observer variability and potentially reducing contouring time. Nonetheless, the role of the physician still remains irreplaceable. This is also confirmed by Meillan et al. [20] in a comparison study between two different automated segmentation softwares (iPlan and Smart Segmentation) applied for hypofractionated radiotherapy for intracranial lesions. The authors observed a substantial equivalency of both softwares, nevertheless highlighting the need of manual editing to perform the best tailored contouring.

A recent review by Francolini et al. [21] collected the literature experiences reporting preliminary studies of artificial intelligence applied to all phases of radiation treatment planning. Particularly in the segmentation process, the authors reported the use of Dice Similarity Coefficient as the most frequently used tool to test the reliability of several auto-segmentation softwares. Despite most of the studies favorably report artificial intelligence systems for automatic contouring for both targets and OARs, some authors also raise concerns due to the need to manually revise and edit automatically generated contours [22].

Further steps forward are awaited from the implementation of deep learning frameworks and artificial intelligence in daily clinical activity, for which some preliminary experiences have been recently published [23–25], supporting the introduction of this technological aids for refining the accuracy in target and OAR identification.

Several advantages might be provided by the introduction of artificial intelligence systems, as machine-learning based networks are supposed to ease the radiation treatment planning procedure in all its phases. The contouring process might be improved both in terms of time-consumption issues and inter-observer variability, as already reported [26].

This study has some limitations; first of all, the small sample size may affect the statistical power of our analysis. Nonetheless, we included three different physicians to better estimate the potential inter-observer variations: the main aim of the present study was to assess the impact of Smartbrush software compared to manual contouring in order to reduce inter-observer variability,A further analysis with a larger sample, other metrics tools, and the assessment of time consumption issues is currently ongoing at our Department.

Moreover, in the specific setting of spinal metastases SBRT, to the best of our knowledge, the use of automated software for treatment planning and target contouring was previously investigated only in our preliminary institutional experience [12], supporting the use of Elements as a safe and effective tool for the clinician.

Long term follow-up data are warranted to further assess the favorable impact of this technology from a clinical perspective in terms of both toxicity and clinical outcomes.

## Conclusions

The Elements® Spine SRS dedicated software for linac-based spinal SBRT treatment is a useful approach for patients with spinal metastases, improved by a dedicated automatic contouring tool (SmartBrush) that allows greater precision and reduction of inter-observer differences in the delineation of the target in SBRT spines.

## Data Availability

The patient information may be shared under ‘IRCCS Sacro cuore – Don Calabria’ hospital IRB approval of amendment on a case by case base. The Element™ planning package is proprietary (BrainLab AG, Munich, Germany) due to patent protection.
